# Predicting High-Grade Acute Urinary Toxicity and Lower Gastrointestinal Toxicity After Postoperative Volumetric Modulated Arc Therapy for Cervical and Endometrial Cancer Using a Normal Tissue Complication Probability Model

**DOI:** 10.3390/curroncol32010026

**Published:** 2025-01-01

**Authors:** Tianyu Yang, Zhe Ji, Runhong Lei, Ang Qu, Weijuan Jiang, Xiuwen Deng, Ping Jiang

**Affiliations:** Department of Radiation Oncology, Peking University Third Hospital, 49 North Garden Road, Haidian District, Beijing 100191, China

**Keywords:** radiotherapy, urinary toxicity, gastrointestinal toxicity, cervical cancer, endometrial cancer

## Abstract

(1) Background: Volumetric modulated arc therapy (VMAT) can deliver more accurate dose distribution and reduce radiotherapy-induced toxicities for postoperative cervical and endometrial cancer. This study aims to retrospectively analyze the relationship between dosimetric parameters of organs at risk (OARs) and acute toxicities and provide suggestions for the dose constraints. (2) Methods: A total of 164 postoperative cervical and endometrial cancer patients were retrospectively analyzed, and the endpoints were grade ≥ 2 acute urinary toxicity (AUT) and acute lower gastrointestinal toxicity (ALGIT). The normal tissue complication probability (NTCP) model was established using the logistic regression model. Restricted cubic spline (RCS) curves were used to explore the association between dosimetric parameters and toxicities. The receiver operating characteristic (ROC) curve, calibration curve, Akaike’s corrected information criterion (AICc), decision curve analysis (DCA), and clinical impact curve (CIC) were analyzed to evaluate the performance of NTCP models. (3) Results: Bladder V_40Gy_ was identified to develop the NTCP model of AUT, and the mean AUC was 0.69 (CI: 0.58–0.80). Three candidate predictors, namely the small intestine V_30Gy_, colon D_45%_, and rectum D_55%_, were identified to develop the NTCP model of ALGIT, and the mean AUC was 0.71 (CI: 0.61–0.80). Both models were considered to have relatively good discriminative accuracy and could provide a high net benefit in clinical applications. (4) Conclusions: We developed NTCP models to predict the probability for grade ≥ 2 AUT and ALGIT. We recommend that bladder V_40Gy_, the small intestine V_30Gy_, colon D_45%_, and rectum D_55%_ be controlled below 42%, 20.4%, 16.9 Gy, and 32.0 Gy, respectively.

## 1. Introduction

Cervical cancer and endometrial cancer are both worldwide health problems. Among them, the incidence of cervical cancer ranks fourth among female malignant tumors worldwide [1]. For endometrial cancer, its incidence ranks sixth, and its overall incidence has gradually increased in the past 30 years, with a tendency towards younger age [2]. Radiotherapy is an important option in the principal treatment for both postoperative cervical and endometrial cancer patients. With the continuous development of radiation physics and medical imaging, the technology of external beam radiotherapy (EBRT) has progressed, and the prognosis of cervical and endometrial cancer continues to improve. However, the toxicities followed by radiotherapy cannot be completely avoided. The occurrence of toxicities may cause patients to suspend treatment and prolong the treatment time, which is detrimental to pelvic control [3]. AUT and ALGIT could also lead to a decline in quality of life and an increase in the incidence of late urinary toxicity and late gastrointestinal toxicity. VMAT is a novel form of IMRT. Compared to VMAT, IMRT requires multiple fixed-angle beams, and it may result in longer treatment delivery time [4]. Furthermore, a large number of monitor units (MUs) are applied during IMRT, which may increase radiation received by OARs, resulting in increased acute and chronic toxicities [5]. VMAT involves a dynamic multileaf collimator, one gantry rotation, a variable dose rate, and gantry speed, which significantly shortens treatment time, reduces inter-fraction setup errors, and improves treatment accuracy [6]. Several studies have reported that, compared with conventional IMRT, VMAT enables significant dose reduction to OARs, fewer MUs, and enhanced plan quality [7,8,9]. Considering that there are limited data available on dose constraints of OARs for postoperative cervical and endometrial cancer with VMAT, it is necessary to further explore the relationship between dosimetric parameters and toxicities to reduce the incidence of AUT and ALGIT.

This study aims to retrospectively analyze the relationship between dosimetric parameters of OARs and acute toxicities and provide suggestions for the dose constraints in VMAT plan design for postoperative cervical and endometrial cancer.

## 2. Materials and Methods

### 2.1. Patients

This study retrospectively reviewed patients with pathologically confirmed cervical or endometrial cancer who underwent radical hysterectomy and VMAT between May 2021 and January 2024 in our institution. The 2018 International Federation of Gynecology and Obstetrics (FIGO) system was used for the clinical stage of cervical cancer, and the 2009 FIGO was used for the clinical stage of endometrial cancer [10,11]. This retrospective study was approved by the Clinical Research Ethics Committee of Peking University Third Hospital (M2023430).

### 2.2. Radiotherapy

For postoperative cervical cancer, EBRT was performed based on postoperative pathological staging, with high-risk factors determined according to the Peters criteria and intermediate-risk factors according to the Sedlis criteria [12,13]. For endometrial cancer, EBRT was performed for patients defined as intermediate–high risk and high risk according to the European Society for Medical Oncology (ESMO) risk classification [14]. Patients were treated with either 45 Gy in 25 fractions or 50.4 Gy in 28 fractions using 6 MV X-rays delivered by a Trilogy linear accelerator (Varian Medical Systems, Palo Alto, CA, USA) with VMAT. Patients with positive surgical margins or lymph node metastasis were treated with 50.4 Gy in 28 fractions and a simultaneous boost with 60 Gy in 28 fractions. In addition, considering that the prognoses of cervical cancer and endometrial cancer are different, postoperative cervical cancer patients were routinely delivered 12 Gy in 2 fractions of high-dose-rate (HDR) brachytherapy, while postoperative endometrial cancer patients were routinely delivered 18 Gy in 3 fractions. Patients were treated with ^192^Ir HDR CT-guided adaptive brachytherapy using single-channel cylinders.

Patients were instructed to empty the rectum as much as possible before positioning, fast for 4 hours, and maintain a fasting state. Additionally, patients were required to empty the bladder and drink 1000 mL of water within 10 minutes to fill the bladder, then hold urine. Positioning was performed when the patient experienced a noticeable urge to urinate. This preparation process was repeated before each radiotherapy fraction. All target areas were delineated by experienced radiation oncologists. The principles of EBRT for delineating target areas for both postoperative cervical and endometrial cancer were the same. The clinical target volume (CTV) included the cervical stump, upper vagina, and lymph nodes (common iliac, external and internal iliac, obturator, and presacral nodes). Patients with positive para-aortic lymph nodes were treated with extended field radiotherapy. The planning target volume (PTV) was defined as CTV with a 3D isotropic 7 mm expansion added to cover the motion of the target. The contours of the OARs were delineated based on the external boundaries as determined by CT simulation. The OARs were delineated according to RTOG guidelines, except that if the CTV covered the intestines, we appropriately trimmed along the intestinal loop during delineation [15]. Dose limitations for OARs included the following: 50% of bladder volume might not receive >40 Gy; 40% of small intestine might not receive >30 Gy, and the maximum dose of the small intestine was 53 Gy; and 50% of rectal volume might not receive >40 Gy, and the maximum dose of the colon was 54 Gy. Cone beam computed tomography (CBCT) was used for image guidance during treatment to ensure accurate positioning and determine the impact of bladder and rectal filling.

VMAT plans were developed by experienced radiation oncologists. Specifically, 95% of PTV was required to be irradiated at 100% of the prescribed level. Considering the movement of OARs, 2–3 mm margins were applied to define the planning organ at risk volume (PRV). When conflicts arose between PTV coverage and the dose constraints of OARs, our approach was to prioritize achieving adequate PTV coverage first. Once satisfactory target coverage was attained, multiple rounds of optimization were performed to minimize the radiation dose to OARs. During this process, exceeding the dose constraints for OARs was permitted. However, in specific cases, such as when a patient developed severe intestinal adhesions after a radical hysterectomy that fell into the pelvis, we would make appropriate adjustments, sacrificing PTV coverage when necessary. The principles of HDR brachytherapy for delineating target areas were as follows. The CTV was delineated by uniformly extending 3 mm from the applicator surface. The treatment length ranged from 3 to 5 cm from the vaginal apex and did not exceed two-thirds of the total vaginal length in a shortened postoperative vagina. The CT-guided brachytherapy planning was performed in the brachytherapy treatment planning system (Varian Medical Systems) and delivered using the Varian GammaMed Plus iX HDR afterloader (Varian Medical Systems).

### 2.3. Chemotherapy

For cervical cancer, according to the National Comprehensive Cancer Network (NCCN) guidelines, patients with high-risk factors were recommended to be delivered cisplatin (40 mg/m^2^) weekly based on concurrent chemotherapy in our institution.

For endometrial cancer, based on the patient’s postoperative pathological staging, pathological type, and postoperative pathological high-risk factors, the patient situation was discussed by the gynecological oncology multi-disciplinary team in our institution. After the discussion, patients first received 2 to 4 cycles of albumin paclitaxel (175 mg/m^2^) combined with cisplatin (75 mg/m^2^) or carboplatin (AUC = 5) before undergoing radiotherapy and then received 2 cycles of chemotherapy after the radiotherapy was completed, i.e., a total of 4 to 6 cycles.

### 2.4. Toxicity Evaluation

All patients were routinely evaluated for their lower gastrointestinal symptoms and urinary symptoms in the outpatient setting before starting radiotherapy. Afterwards, AUT, which included urinary frequency, urinary urgency, urinary pain, hematuria, increased nocturia, and acute urinary retention, and ALGIT, which included diarrhea, bloody stool, intestinal obstruction, intestinal perforation, intestinal fistula, and colicky abdominal pain, were evaluated and recorded through outpatient visits or telephone follow-ups every week during and 1 week after radiotherapy. Subsequently, AUT and ALGIT were evaluated and recorded every month within 3 months after completing radiotherapy. These AUT and ALGIT were noted and graded according to the RTOG acute radiation morbidity scoring criteria. The detailed definitions of AUT and ALGIT are shown in Table S1. The highest grades of AUT and ALGIT observed during follow-ups were analyzed.

### 2.5. Dosimetric Parameters Extraction

The relative volumes receiving x Gy (V_xGy_ [%]) and the dose in x% of the OAR volume (D_x%_ [Gy]) were extracted from dose–volume histograms (DVH). The V_xGy_ [%] in 5 Gy intervals (within 5–55 Gy) and D_x%_ [Gy] in 5% intervals (within 5–95%) were extracted. In addition, the dose in 2 cm^3^ of the OAR volume (D_2cm_^3^ [Gy]), the total volume of OAR, mean dose (D_mean_ [Gy]), maximum dose (D_max_ [Gy]), and minimum dose (D_min_ [Gy]) were also extracted. Only the DVH parameters in the EBRT plan were extracted from the Eclipse planning system and analyzed.

### 2.6. The NTCP Model

To predict the probability of AUT and ALGIT, we established NTCP models that were based on logistic regression and followed the Transparent Reporting of a multivariable prediction model for Individual Prognosis Or Diagnosis (TRIPOD) statement [16]. Furthermore, considering that our sample size is relatively small, we did not divide the samples, and the prediction model followed a type 1b TRIPOD model.

The models were established with the following steps. Firstly, considering the collinearity between candidate parameters, LASSO regression was performed for candidate parameter selection from the clinical and dosimetric characteristics, which would avoid model overfitting and multivariate collinearity. The optimal penalty value (λ) was determined by the one standard error of the minimum criteria (1-SE criteria) selected by 3-fold internal cross-validation. Secondly, if there were several parameters selected by LASSO regression, multivariable logistic regression analysis was used to further identify the predictors; the method was backward stepwise, and *p*-value > 0.1 was excluded. Thirdly, the NTCP model was established using the logistic regression model, and the formula was as follows:

(1) $$\mathrm{NTCP}=\frac{1}{\begin{array}{c} 1+e^{-(\beta_{0}+\beta_{1x1}+\beta_{2x2}+\cdots +\beta_{nxn})} \end{array}}$$

where x1, x2 … is the final candidate predictor, and β1, β2 … is the regression coefficient of the corresponding final candidate predictor. The performance of the NTCP models was evaluated based on the following methods. We generated the ROC curve and calculated each model’s mean AUC using the bootstrap resampling method with 1000 replicates to evaluate discriminative ability. The AUC value was used to quantify the discriminative ability, with 0.5 denoting bad discrimination and 1.0 denoting excellent discrimination. The higher the value of the AUC, the better the performance of the model. The calibration curve was generated based on the bootstrap resampling method with 1000 replicates to assess the calibration. The vertical axis of the curve denoted the observed probability, while the horizontal axis denoted the predicted probability. The closer to the reference line of 45°, the better the perfectly accurate prediction of the model. We assessed the prediction performance using AICc, which considered the model deviance as well as the sample size and number of estimated parameters in the model. The lower the value of the AICc, the better the forecast performance. A DCA curve was generated to evaluate whether the model improved clinical decision-making and show the net benefit at a range of clinically reasonable risk thresholds. CIC was generated to further assess the net benefit of interventions at various threshold probabilities. Finally, RCS curves were used to explore the association between dosimetric parameters and acute toxicities.

The IBM SPSS Statistics 26.0 and software R (version 4.3.0) were used for the statistical analysis and to plot the curves.

## 3. Results

### 3.1. Patients

Between May 2021 and January 2024, a total of 187 postoperative patients with a minimum 3-month follow-up were included in this study. In total, 6 patients were excluded because of the absence of clinical examinations, and 17 were excluded because of loss to follow-up. A total of 164 postoperative patients were eligible. Table 1 shows the clinical characteristic parameters and the incidence of AUT. As can be seen, 96 (58.5%) patients experienced grade 0 AUT, 38 (23.2%) patients experienced grade 1 AUT, 28 (17.1%) patients experienced grade 2 AUT, and 2 (1.2%) patients experienced grade 3 AUT during treatment. No grade 4 AUT was observed. Table 2 shows the clinical characteristic parameters and the incidence of ALGIT. As can be seen, 83 (50.6%) patients experienced grade 0 ALGIT, 47 (28.7%) patients experienced grade 1 ALGIT, 31 (18.9%) patients experienced grade 2 ALGIT, and 3 (1.8%) patients experienced grade 3 ALGIT during treatment. No grade 4 ALGIT was observed. The results of univariable analyses of the clinical characteristic parameters associated with grade ≥ 2 AUT are shown in Table 3. The results of univariable analyses of the clinical characteristic parameters associated with grade ≥ 2 ALGIT are shown in Table 4. All clinical characteristic parameters have a *p*-value > 0.05. The median follow-up time was 19 months. Among the participants, three patients experienced RTOG grade 1 chronic urinary toxicity, three patients experienced RTOG grade 2 chronic urinary toxicity, and one patient experienced RTOG grade 3 urinary toxicity. Five patients experienced RTOG grade 1 chronic urinary toxicity, three patients experienced RTOG grade 2 chronic urinary toxicity, and two patients experienced RTOG grade 3 chronic urinary toxicity. Dosimetric parameters of the PTV was shown in Table S4.

### 3.2. The NTCP Model: AUT

**Figure 1:**
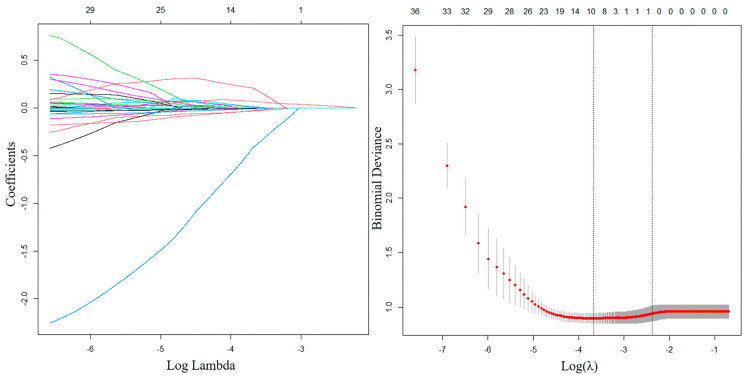
A coefficient plot produced against the log (λ) sequence is shown in the left figure. The right figure provides variable selection using the LASSO binary logistic regression model’s penalty parameter λ and 3-fold cross-validation via the minimum criteria. The optimal penalty value (λ) was 0.0926. Dotted vertical lines are drawn at the optimal values by the minimum criteria and the 1 standard error of the minimum criteria (the 1-SE criteria).

The results of univariable analyses of dosimetric parameters in patients without and with grade ≥ 2 AUT are shown in Table S2. The result of LASSO regression is shown in Figure 1. The optimal penalty value (λ) was 0.0926, and only bladder V_40Gy_ was identified as the final candidate predictor. The NTCP model of AUT was established as follows:

$$I.\mathrm{NTCP}=\frac{1}{\begin{array}{c} 1+e^{-(0.070*Bladder\;V40Gy-5.087)} \end{array}}$$

**Figure 2:**
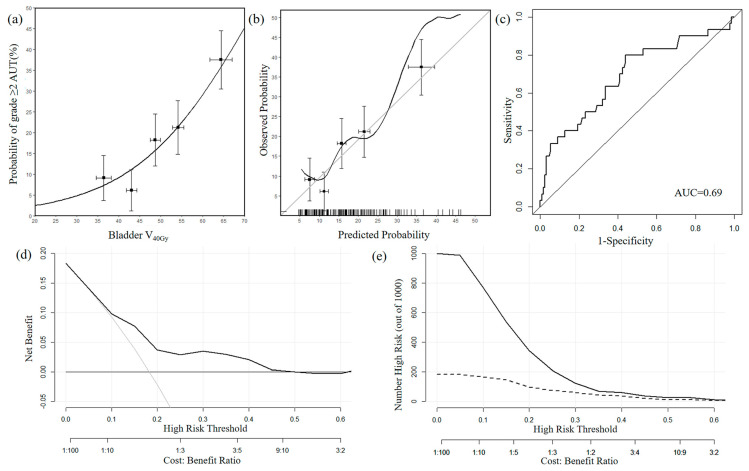
(**a**) Model of AUT. The points with error bars indicate observed normal tissue complication probability values with their standard deviation. (**b**) Corresponding calibration plots and curves of AUT. (**c**) Receiver operating characteristic curve of AUT. (**d**) Decision curve analysis of AUT. (**e**) Clinical impact curve of AUT.

We calculated that β0 was −5.087 (CI: −8.231 to −2.937) and β1 was 0.070 (CI: 0.029 to 0.124). The NTCP model, the calibration curve, and the ROC curve are shown in Figure 2a–c.

The mean AUC was 0.69 (CI: 0.58–0.80), which was considered to have relatively good discriminative accuracy. The DCA curve and the CIC are shown in Figure 2d,e. On the DCA curve and CIC, within a threshold probability range of 10% to 50%, using this predictive model to predict the probability of grade ≥ 2 AUT can lead to net clinical benefits. Based on the predictive model of AUT, we calculated that bladder V_40Gy_ at the predicted 10% grade ≥ 2 AUT probability was 42%.

### 3.3. The NTCP Model: ALGIT

**Figure 3:**
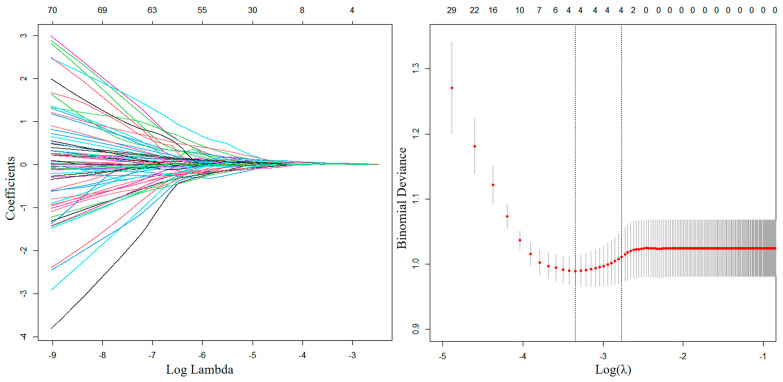
A coefficient plot produced against the log (λ) sequence is shown in the left figure. The right figure provides variable selection using the LASSO binary logistic regression model penalty parameter λ and 3-fold cross-validation via the minimum criteria. The optimal penalty value (λ) was 0.0628. Dotted vertical lines are drawn at the optimal values by the minimum criteria and the 1 standard error of the minimum criteria (the 1-SE criteria).

The results of univariable analyses of dosimetric parameters in patients without and with grade ≥ 2 ALGIT are shown in Table S3. The result of LASSO regression is shown in Figure 3. The optimal penalty value (λ) was 0.0628, and four candidate predictors, namely the small intestine V_30Gy_, colon D_15%_, colon D_45%_, and rectum D_55%_, were identified. Considering that the Spearman correlation coefficient was >0.5 between colon D_15%_ and colon D_45%_, we defined the group including the small intestine V_30Gy_, colon D_15%_, and rectum D_55%_ as Group A and the group including the small intestine V_30Gy_, colon D_45%_, and rectum D_55%_ as Group B. Multivariable logistic regression analysis was then used; the method was backward stepwise, and *p*-value > 0.1 was excluded. The results are shown in Table 5. Considering that the *p*-value of colon D_15%_ was >0.1 in Group A, we established two models, one of which included colon D_45%_ and the other did not. A total of three multivariate NTCP models were established as follows:

$$\mathrm{II}.\mathrm{NTCP}=\frac{1}{\begin{array}{c} 1+e^{-(0.037*Small\;Intestine\;V30Gy+0.121*RectumD55\%-8.099)} \end{array}}$$

$$\mathrm{III}.\mathrm{NTCP}=\frac{1}{\begin{array}{c} 1+e^{-(0.034*Small\;Intestine\;V30Gy+0.060*RectumD55\%+0.099*ColonD15\%-9.412)} \end{array}}$$

$$\mathrm{IV}.\mathrm{NTCP}=\frac{1}{\begin{array}{c} 1+e^{-(0.039*Small\;Intestine\;V30Gy+0.069*RectumD55\%+0.072*ColonD45\%-7.439)} \end{array}}$$

**Figure 4:**
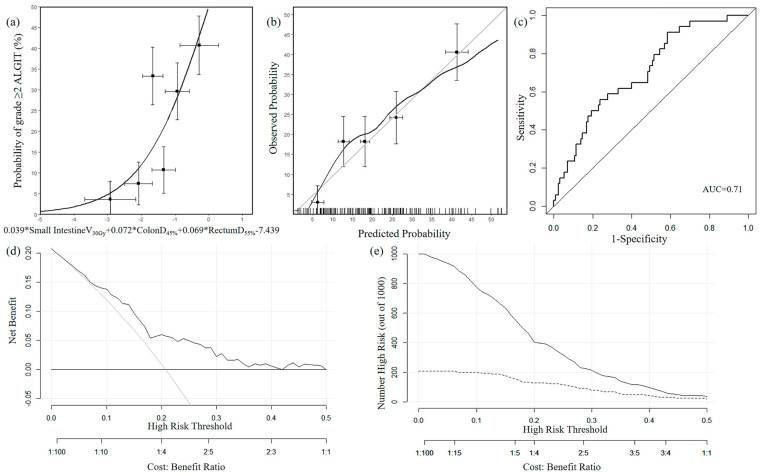
(**a**) Model of ALGIT. The points with error bars indicate observed normal tissue complication probability values with their standard deviation. (**b**) Corresponding calibration plots and curves of ALGIT. (**c**) Receiver operating characteristic curve of ALGIT. (**d**) Decision curve analysis of ALGIT. (**e**) Clinical impact curve of ALGIT.

The performance of these models is shown in Table 5. Model III and Model IV had the same mean AUC and were both higher than Model II. To improve the prediction ability, based on the AICc values, we ultimately chose Model IV as the final model. Model IV, the calibration curve, and the ROC curve are shown in Figure 4a–c. The calibration curve showed relatively satisfactory agreements between the predicted probability and the actual observed probability. The mean AUC was 0.71 (CI: 0.61–0.80), which was considered to have good discriminative accuracy. The DCA curve and the CIC are shown in Figure 4d,e. On the DCA curve and CIC, these curves showed that within a threshold probability range of 5% to 40%, the NTCP model demonstrated an overall net benefit, suggesting high clinical potential.

**Figure 5:**
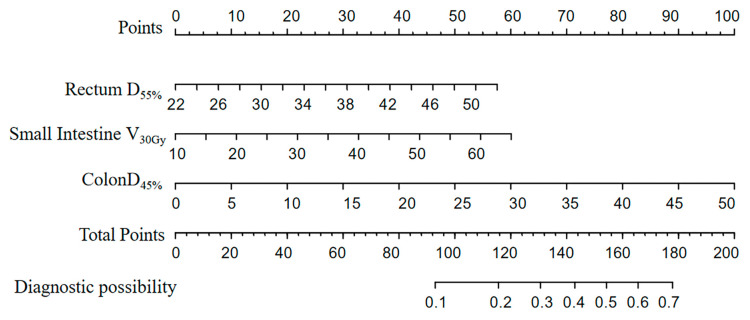
Nomogram to predict the probability for grade ≥ 2 ALGIT for patients with cervical or endometrial cancer who underwent radical hysterectomy and VMAT.

**Figure 6:**
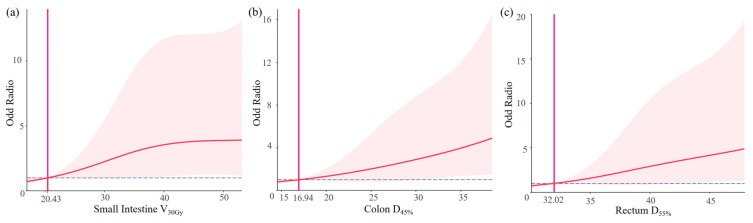
(**a**) Restricted cubic spline of the small intestine V_30Gy_ and ALGIT. (**b**) Restricted cubic spline of colon D_45%_ and ALGIT. (**c**) Restricted cubic spline of rectum D_55%_ and ALGIT.

The graphic nomogram is shown in Figure 5. Figure 6 shows the RCS curve of each final candidate predictor. RCS analyses further indicated the cut-off of the small intestine V_30Gy_, colon D_45%_, and rectum D_55%_ were 20.4%, 16.9 Gy, and 32.0 Gy, respectively.

## 4. Discussion

Acute toxicities are common issues in the radiotherapy of postoperative cervical and endometrial cancer, which could impact quality of life and treatment completion [3]. Especially for patients who receive radical hysterectomy, a significant portion of the small intestine falls into the vacated space in the pelvis, thereby increasing the volume of small intestine that receives a high dose. Similarly, the bladder would move towards the target, and a large portion of the bladder is included in the field of radiotherapy [17,18,19]. Compared to conventional IMRT, VMAT could significantly shorten treatment time, improve treatment accuracy and dose distribution, and provide possibilities for better protection of OARs. The main objective of this study was to develop a predictive model for AUT and ALGIT.

In our study, there was no significant difference between AUT or ALGIT and clinical characteristics. Vandecasteele et al. found that para-aortic lymph node irradiation significantly increased the incidence of grade ≥ 2 radiation-related toxicities [20]. On the contrary, Luo et al. prospectively compared 129 postoperative patients and demonstrated that para-aortic lymph node irradiation did not significantly increase the incidence of grade ≥ 2 acute gastrointestinal toxicity [21]. In our study, extended field radiotherapy showed an increasing trend in grade ≥ 2 AUT and ALGIT, but no significant difference was found. However, the sample size of patients receiving extended field radiotherapy in our study was relatively small, and further large-scale randomized clinical trials are needed to explore the relationship between extended field radiotherapy and acute toxicities.

Regarding the NTCP model of AUT, bladder V_40Gy_ was identified as the final candidate predictor, and the majority of bladder V_40Gy_ values were in the interval of 10% to 30%. On the calibration curve, when the value of bladder V_40Gy_ was between 10% and 30%, the calibration curve was closer to the reference line, suggesting the accurate prediction of the model. However, when the value of bladder V_40Gy_ was less than 10% and greater than 30%, there was a deviation between the calibration curve and the reference line, which was possibly caused by a few extreme values within this range in the exploration cohort. Vandecasteele et al. found that concurrent chemotherapy significantly increased the incidence of grade ≥ 2 AUT, which was mainly manifested by an increase in nocturia [20]. However, there was no significant statistical difference between concurrent chemotherapy and grade ≥ 2 AUT in our study, which was caused by hydration and subsequent dehydration at night. In our institution, considering that most of the patients were elderly people, we usually gave mannitol for dehydration after the completion of chemotherapy to avoid excessive fluid load. This might explain the fact that although patients received the same chemotherapy regime, we did not observe increased nocturia and significant differences in grade ≥ 2 AUT. Recent studies have assessed bladder spatial dose parameters through a pixel-wise method for analysis of bladder dose–surface maps. They demonstrated that there were differences in radiation tolerance between sub-structures, with the bladder trigone being more sensitive to radiation than the other sub-structures, and a reduction of the dose in the bladder trigone might significantly reduce AUT or late urinary toxicities [22,23]. However, in clinical practice, it is difficult to identify and delineate the bladder trigone, and the bladder trigone is almost within the target for patients receiving radical hysterectomy. Therefore, it is necessary to further explore constraint dosimetric parameters. Compared with the NCCN guidelines, which recommend that V_35%_ of the bladder volume should not receive >45 Gy, we provide further recommendations for the bladder dose constraint [24,25].

Regarding the NTCP model of ALGIT, the small intestine V_30Gy_, the colon D_45%_, and the rectum D_55%_ were identified as final candidate predictors. Several studies have reported dosimetric predictors for acute or late gastrointestinal toxicity. Isohashi et al. analyzed 62 postoperative patients with 9% grade > 2 gastrointestinal toxicity using the Common Terminology Criteria for Events version 4.0. They showed that V_40Gy_ and V_45Gy_ of the small bowel loops were predictive for the development of both acute and chronic gastrointestinal toxicity [26]. Simpson et al. analyzed 55 patients with 46% grade > 2 gastrointestinal toxicity using the RTOG scoring system. They showed that the small intestine V_45Gy_ was the predictor for acute gastrointestinal toxicity [27]. The dosimetric predictor reported by studies is not consistent, which is possibly caused by small intestine motion and a discrepancy between the planned dose and the actual dose.

Several studies have demonstrated that concurrent chemotherapy would add to radiotherapy-induced acute gastrointestinal toxicity [28,29]. In our study, compared to those patients without any chemotherapy, the incidence of ALGIT among patients who underwent concurrent chemotherapy or adjuvant therapy increased, but it was not statistically significant. The chemotherapy regimens in our study were mainly cisplatin and paclitaxel. The untoward reactions caused by cisplatin are nausea, vomiting, mild to moderate myelosuppression, nephrotoxicity, and neurotoxicity [30]. Similarly, the untoward reactions caused by paclitaxel are mainly hair loss, neurotoxicity, myelosuppression, nausea, and vomiting. Both cisplatin and paclitaxel could lead to lower gastrointestinal symptoms, such as diarrhea and abdominal pain, but compared to the frequency of the symptoms from the upper and lower gastrointestinal tract, we speculate that chemotherapy mainly caused the upper gastrointestinal toxicity. A quantitative analysis of normal tissue effects in the clinic (QUANTEC) report found that, whether it is radiation-induced injuries to the rectum, the small intestine, or the colon, symptoms could manifest as diarrhea and abdominal pain [28,31]. The symptoms of different OARs caused by radiotherapy lack specificity. Furthermore, it is difficult to accurately determine the location of radiation injury based solely on symptoms in clinical practice. Therefore, we used the RTOG scoring system to evaluate ALGIT, which treats the lower gastrointestinal tract as a whole.

In our institution, brachytherapy was generally added to the EBRT after 20 fractions. The majority of patients reached peak toxicities of ALGIT in week 3 and week 4 of treatment and reached peak toxicities of AUT in week 4 of treatment, at which point brachytherapy had not yet been delivered. Khalid et al. showed that most patients reached peak toxicity in week 3 and week 4 of treatment, which is in concordance with our results [32]. Therefore, although the acute toxicities were collected up to three months after treatment, we think that EBRT played a dominant role in acute toxicities, and only the DVH parameters in the EBRT plan were extracted and analyzed. However, this view needs further research to confirm.

The RCS curves were utilized to explore the association between dosimetric predictors and toxicities and the cut-off values of dosimetric parameters. On the RCS curves, we calculated that the cut-off values of the small intestine V_30Gy_, colon D_45%_, and rectum D_55%_ were 20.4%, 16.9 Gy, and 32.0 Gy, respectively. Compared to the dose constraints recommended by the current guidelines, although the dose constraints we provided were stricter, VMAT’s better dose distribution could provide the possibility for our dose constraints reducing the toxicities.

In conclusion, the generated NTCP model, along with the dose constraint, could be useful in clinical practice to reduce acute toxicities. With the application of this model and the dose constraint, physicians could further optimize the VMAT plan and dosimetric parameters, and clinicians can easily identify patients with a high risk of AUT and ALGIT and use early intervention to reduce the incidence of AUT and ALGIT. However, further validation of our results should be conducted in different centers using prospective cohorts.

### Limitation

This study had some limitations. First, the DVH parameters were not converted into equivalent doses in 2 Gy. Second, to improve the quality of life of patients and further reduce the occurrence of toxicities, we considered grade 2 as “high-grade” toxicity, which may lead us to propose stricter dose constraints. Further studies are needed to validate our results. Third, our sample size was relatively small, and the model was developed using data from a single-center cohort. Additionally, the model was based on a retrospective cohort study. Prospective cohorts are needed in future studies for further validation.

## 5. Conclusions

We developed NTCP models to predict the probability for grade ≥ 2 AUT and ALGIT. We recommend that bladder V_40Gy_, the small intestine V_30Gy_, colon D_45%_, and rectum D_55%_ be controlled below 42%, 20.4%, 16.9 Gy, and 32.0 Gy, respectively. Based on this model, we could further intervene in high-risk populations and provide a basis for the development of individualized radiotherapy plans.

## Figures and Tables

**Table 1:** Comparison of clinical characteristic parameters and the incidence of AUT.

	All	Grade < 2	Grade ≥ 2	*p*	Cervical Cancer	Endometrial Cancer	*p*
n (%)	164 (100)	134 (81.7)	30 (18.3)		87 (53.0)	77 (47.0)	
Age (years), medium (range)	56 (26–79)	56 (26–79)	57 (33–77)	0.475	55 (31–77)	54 (26–79)	0.125
Height (cm), medium (range)	160 (150–172)	160 (150–172)	160 (153–172)	0.901	160 (150–170)	160 (150–172)	0.316
Weight (kg), medium (range)	60 (37–85)	59 (37–85)	61 (50–83)	0.397	60 (37–85)	62 (40–83)	0.724
BMI (kg/m^2^), medium (range)	23.6 (14.5–34.0)	23.5 (14.5–34.0)	24.1 (19.1–29.1)	0.136	23.4 (14.5–34.0)	23.9 (16.0–32.5)	0.996
Pathology, n (%)				0.340			**<0.001**
Cervical cancer	87 (53.0)	74 (55.2)	13 (43.3)		87 (100.0)	0 (0.0)	
Squamous cell carcinoma	77 (47.0)	62 (46.3)	15 (50.0)		77 (88.5)	0 (0.0)	
Adenocarcinoma	8 (4.9)	7 (5.2)	1 (3.3)		8 (9.2)	0 (0.0)	
Adenosquamous carcinoma	2 (1.2)	2 (1.5)	0 (0.0)		2 (2.3)	0 (0.0)	
Endometrial cancer	77 (47.0)	60 (44.8)	17 (56.7)		0 (0.0)	77 (100.0)	
Endometrioid carcinoma	62 (37.8)	54 (40.3)	8 (26.7)		0 (0.0)	62 (80.5)	
Mixed carcinoma	8 (4.9)	5 (3.7)	3 (10.0)		0 (0.0)	8 (10.4)	
Clear cell carcinoma	2 (1.2)	1 (0.7)	1 (3.3)		0 (0.0)	2 (2.6)	
Serous carcinoma	5 (3.0)	3 (2.2)	2 (6.7)		0 (0.0)	5 (6.5)	
Clinical stage (FIGO), n (%)				0.584			**0.005**
I	80 (48.8)	67 (50.0)	13 (43.3)		42 (48.3)	38 (49.4)	
II	39 (23.8)	31 (23.1)	8 (26.7)		28 (32.2)	11 (14.3)	
III	40 (24.4)	33 (24.6)	7 (23.3)		17 (19.5)	23 (29.9)	
IV	5 (3.0)	3 (2.2)	2 (6.7)		0 (0.0)	5 (6.5)	
EBRT dose (Gy), n (%)				0.681			0.175
45.0	39 (23.8)	31 (23.1)	8 (26.7)		17 (19.5)	22 (28.6)	
50.4	125 (76.2)	103 (76.9)	22 (73.3)		70 (80.5)	55 (71.4)	
Chemotherapy, n (%)				0.096			**<0.001**
No	53 (32.3)	41 (30.6)	12 (40.0)		26 (29.9)	27 (35.1)	
Adjuvant chemotherapy	50 (30.5)	38 (28.4)	12 (40.0)		0 (32.3)	50 (64.9)	
Concurrent chemotherapy	61 (37.2)	55 (41.0)	6 (20.0)		61 (70.1)	0 (0.0)	
HDR brachytherapy, n (%)				0.167			0.611
Yes	95 (57.9)	81 (60.4)	14 (46.7)		52 (59.8)	43 (55.8)	
No	69 (42.1)	53 (39.6)	16 (53.3)		35 (40.2)	34 (44.2)	
KPS, n (%)				0.700			0.204
80	11 (6.7)	10 (7.5)	1 (3.3)		3 (3.4)	8 (10.4)	
90	50 (30.5)	41 (30.6)	9 (30.0)		28 (32.2)	22 (28.6)	
100	103 (62.8)	83 (61.9)	20 (66.7)		56 (64.4)	47 (61.0)	
Extended field radiotherapy, n (%)				0.751			0.055
Yes	14 (8.4)	11 (8.2)	3 (10.0)		4 (4.6)	10 (13.0)	
No	150 (91.5)	123 (91.8)	27 (90.0)		83 (95.4)	67 (87.0)	

Note: *p* value is derived from the chi-square test in nominal variables and independent-sample *t* test in continuous variables. Statistically significant *p*-values are marked in bold. Abbreviations: BMI: body mass index; KPS: Karnofsky performance score; EBRT: external beam radiotherapy; FIGO: International Federation of Gynecology and Obstetrics.

**Table 2:** Comparison of clinical characteristic parameters and the incidence of ALGIT.

	All	Grade < 2	Grade ≥ 2	*p*	Cervical Cancer	Endometrial Cancer	*p*
n (%)	164 (100)	130 (79.3)	34 (20.7)		87 (53.0)	77 (47.0)	
Age (years), medium (range)	56 (26–79)	56 (26–79)	56 (31–67)	0.915	55 (31–77)	54 (26–79)	0.125
Height (cm), medium (range)	160 (150–172)	160 (150–172)	159 (152–167)	0.203	160 (150–170)	160 (150–172)	0.316
Weight (kg), medium (range)	60.0 (37.0–85.0)	60.0 (37.0–85.0)	57.5 (43.0–78.0)	0.321	60 (37.0–85.0)	62 (40.0–83.0)	0.724
BMI (kg/m^2^), medium (range)	23.6 (14.5–34.0)	23.7 (14.5–34.0)	23.3 (17.9–29.2)	0.530	23.4 (14.5–34.0)	23.9 (16.0–32.5)	0.996
Pathology, n (%)				0.082			**<0.001**
Cervical cancer	87 (53.0)	67 (51.5)	20 (58.8)		87 (100.0)	0 (0.0)	
Squamous cell carcinoma	77 (47.0)	58 (44.6)	19 (55.9)		77 (88.5)	0 (0.0)	
Adenocarcinoma	8 (4.9)	7 (5.4)	1 (2.9)		8 (9.2)	0 (0.0)	
Adenosquamous carcinoma	2 (1.2)	2 (1.5)	0 (0.0)		2 (2.3)	0 (0.0)	
Endometrial cancer	77 (47.0)	63 (48.5)	14 (41.2)		0 (0.0)	77 (100.0)	
Endometrioid carcinoma	62 (37.8)	51 (39.2)	11 (32.4)		0 (0.0)	62 (80.5)	
Mixed carcinoma	8 (4.9)	7 (5.4)	1 (2.9)		0 (0.0)	8 (10.4)	
Clear cell carcinoma	2 (1.2)	0 (0.0)	2 (5.9)		0 (0.0)	2 (2.6)	
Serous carcinoma	5 (3.0)	5 (3.8)	0 (0.0)		0 (0.0)	5 (6.5)	
Clinical stage (FIGO), n (%)				0.673			**0.005**
I	80 (48.8)	64 (49.2)	16 (47.0)		42 (48.3)	38 (49.4)	
II	39 (23.8)	30 (23.1)	9 (26.5)		28 (32.2)	11 (14.3)	
III	40 (24.4)	31 (23.8)	9 (26.5)		17 (19.5)	23 (29.9)	
IV	5 (3.0)	5 (3.8)	0 (0.0)		0 (0.0)	5 (6.5)	
EBRT dose (Gy), n (%)				0.679			0.175
45.0	39 (23.8)	30 (23.1)	9 (26.5)		17 (19.5)	22 (28.6)	
50.4	125 (76.2)	100 (76.9)	25 (73.5)		70 (80.5)	55 (71.4)	
Chemotherapy, n (%)				0.240			**<0.001**
No	53 (32.3)	46 (35.4)	7 (20.6)		26 (29.9)	27 (35.1)	
Adjuvant chemotherapy	50 (30.5)	37 (28.5)	13 (38.2)		0 (32.3)	50 (64.9)	
Concurrent chemotherapy	61 (37.2)	47 (36.2)	14 (41.2)		61 (70.1)	0 (0.0)	
HDR brachytherapy, n (%)				0.368			0.611
Yes	95 (57.9)	73 (56.2)	22 (64.7)		52 (59.8)	43 (55.8)	
No	69 (42.1)	57 (43.8)	12 (35.3)		35 (40.2)	34 (44.2)	
KPS, n (%)				0.155			0.204
80	11 (6.7)	11 (8.5)	0 (0.0)		3 (3.4)	8 (10.4)	
90	50 (30.5)	37 (28.5)	13 (38.2)		28 (32.2)	22 (28.6)	
100	103 (62.8)	82 (63.1)	21 (61.8)		56 (64.4)	47 (61.0)	
Extended field radiotherapy, n (%)				0.449			0.424
Yes	14 (8.4)	10 (7.7)	4 (11.8)		6 (6.9)	8 (10.4)	
No	150 (91.5)	120 (92.3)	30 (88.2)		81 (93.1)	69 (89.6)	

Note: *p* value is derived from the chi-square test in nominal variables and independent-sample *t* test in continuous variables. Statistically significant *p*-values are marked in bold. Abbreviations: BMI: body mass index; KPS: Karnofsky performance score; EBRT: external beam radiotherapy; FIGO: International Federation of Gynecology and Obstetrics.

**Table 3:** Univariable logistic regression analyses of clinical characteristics for grade ≥ 2 AUT.

Clinical Parameters	OR	CI	*p*	AUC
Age (years)	0.991	0.956–1.027	0.607	0.466
Height (cm)	1.009	0.926–1.100	0.835	0.500
Weight (kg)	1.025	0.983–1.070	0.249	0.571
BMI (kg/m^2^)	1.067	0.951–1.198	0.270	0.577
Type (reference = cervical cancer)			0.241	0.559
Endometrial cancer	1.613	0.726–3.584		
Pathology (reference = squamous cell carcinoma)			0.457	0.521
Adenocarcinoma	0.590	0.067–5.171	0.634	
Adenosquamous carcinoma	0.000	0.000–0.000	0.999	
Endometrioid carcinoma	0.612	0.241–1.556	0.303	
Mixed carcinoma	2.480	0.532–11.55	0.247	
Clear cell carcinoma	4.133	0.244–69.943	0.325	
Serous carcinoma	2.756	0.422–17.986	0.290	
Clinical stage (FIGO) (reference = I)			0.617	0.538
II	1.330	0.500–3.538	0.568	
III	1.093	0.399–2.999	0.863	
IV	3.436	0.522–22.635	0.199	
External beam radiotherapy dose (Gy) (reference = 45)			0.682	0.482
50.4	0.828	0.335–2.043		
Chemotherapy (reference = No)			0.110	0.507
Adjuvant chemotherapy	0.373	0.129–1.076	0.068	
Concurrent chemotherapy	1.079	0.433–2.691	0.871	
HDR brachytherapy (reference = Yes)			0.170	0.431
No	0.573	0.258–1.270		
KPS (reference = 80)			0.713	0.530
90	2.195	0.248–19.391	0.479	
100	2.410	0.291–19.931	0.415	
Extended field radiotherapy (reference = No)			0.753	0.509
Yes	1.096	0.994–1.209		

**Table 4:** Univariable logistic regression analyses of clinical characteristics for grade ≥ 2 ALGIT.

Clinical Parameters	OR	CI	*p*	AUC
Age (years)	1.006	0.975–1.039	0.696	0.399
Height (cm)	1.024	0.943–1.111	0.576	0.521
Weight (kg)	0.986	0.947–1.027	0.505	0.464
BMI (kg/m^2^)	0.951	0.851–1.063	0.375	0.454
Type (reference = cervical cancer)				0.464
Endometrial cancer	0.744	0.347–1.599	0.449	
Pathology (reference = squamous cell carcinoma)			0.952	0.534
Adenocarcinoma	0.59	0.067–5.171	0.634	
Adenosquamous carcinoma	0	0–0	0.999	
Endometrioid carcinoma	1.206	0.531–2.737	0.655	
Mixed carcinoma	1.378	0.253–7.518	0.711	
Clear cell carcinoma	0	0–0	0.999	
Serous carcinoma	2.756	0.422–17.986	0.29	
Clinical stage (FIGO) (reference = I)			0.392	0.559
II	0.788	0.28–2.219	0.652	
III	1.644	0.673–4.013	0.275	
IV	2.889	0.443–18.842	0.268	
External beam radiotherapy dose (Gy) (reference = 45)				0.502
50.4	1.018	0.418–2.476	0.969	
Chemotherapy (reference = No)			0.251	0.585
Adjuvant chemotherapy	1.957	0.724–5.29	0.185	
Concurrent chemotherapy	2.309	0.836–6.375	0.106	
HDR brachytherapy (reference = Yes)				0.543
No	1.432	0.654–3.135	0.37	
KPS (reference = 80)			0.569	0.548
90	0.857	0.155–4.732	0.86	
100	1.367	0.276–6.763	0.701	
Extended field radiotherapy (reference = No)			0.449	0.520
Yes	1.046	0.916–1.194		

**Table 5:** Performance of multivariate logistic regression models with dose–volume parameters.

Model	Parameters	β0 (95% CI)	β1 (95% CI)	*p*	β2 (95% CI)	*p*	β3 (95% CI)	*p*	Mean AUC (95% CI)	AICc
II	1. Small intestine V_30Gy_ 2. Colon D_15%_	−8.099 (−15.544 to −3.634)	0.037 (0.005 to 0.075)	**0.04**	0.121 (0.023 to 0.280)	**0.02**			0.70 (0.60 to 0.78)	161.80
III	1. Small intestine V_30G_y 2. Rectum D_55%_ 3. Colon D_15%_	−9.412 (−17.713 to −4.465)	0.034 (−0.002 to 0.071)	**0.06**	0.060 (−0.020 to 0.149)	0.16	0.099 (0.004 to 0.258)	**0.07**	0.71 (0.60 to 0.80)	161.69
IV	1. Small intestine V_30Gy_ 2. Rectum D_55%_ 3. Colon D_45%_	−7.439 (−17.106 to −4.428)	0.039 (0.001 to 0.071)	**0.03**	0.069 (−0.020 to 0.150)	**0.09**	0.072 (0.006 to 0.250)	**0.02**	0.71 (0.61 to 0.80)	159.34

Note: Mean AUC values and confidence intervals (CI) were obtained using 1000 bootstrap samples. *p*-values and AICc were calculated on the exploration cohort. Statistically significant *p*-values are marked in bold. Abbreviations: AUC: area under the receiver-operating characteristics curve; CI: confidence interval; β0: intercept; β1: regression coefficient of the parameter 1; β2: regression coefficient of the parameter 2; β3: regression coefficient of the parameter 3; D_x%_ [Gy]: the dose in x% of the volume of the organ at risk; AICc = Akaike’s corrected information criterion; V_xGy_ [%]: the relative volumes receiving xGy.

## Data Availability

The datasets generated and/or analyzed during the current study are not publicly available due to patient privacy information and confidentiality policy but are available from the corresponding author upon reasonable request.

## Supplementary Materials

The following supporting information can be downloaded at https://www.mdpi.com/article/10.3390/curroncol32010026/s1, Table S1: The definitions of acute lower gastrointestinal toxicity and acute urinary toxicity according to the Radiation Therapy Oncology Group acute radiation morbidity scoring criteria; Table S2: The results of univariable analyses of dosimetric parameters in patients without and with grade ≥ 2 AUT; Table S3: The results of univariable analyses of dosimetric parameters in patients without and with grade ≥ 2 ALGIT; Table S4: Dosimetric parameters of the PTV.
