# Supplementary material for: Predicting High-Grade Acute Urinary Toxicity and Lower Gastrointestinal Toxicity After Postoperative Volumetric Modulated Arc Therapy for Cervical and Endometrial Cancer Using a Normal Tissue Complication Probability Model
Source: Curr Oncol. 2025 Jan 1;32(1):26. doi: 10.3390/curroncol32010026 (PMC11763444; doi:10.3390/curroncol32010026)
Supplement: Supplementary file 1 [file curroncol-32-00026-s001.zip › curroncol-3346653-supplementary.pdf]

Supplementary Tables

Table S1 The definitions of acute lower gastrointestinal toxicity and acute urinary toxicity according to the Radiation Therapy Oncology Group acute radiation morbidity scoring criteria

Grade	Acute lower gastrointestinal toxicity	Acute urinary toxicity
0	No change	No change
1	Increased frequency or change of in quality of bowel habits not requiring medication; rectal discomfort not requiring analgesics	Frequency of urination or nocturia twice pretreatment habit; dysuria, urgency not requiring medication
2	Diarrhea requiring parasymphatolytic drugs (e.g. diphenoxylate); mucous discharge not necessitating sanitary pads; rectal or abdominal pain requiring analgesics	Frequency of urination or nocturia less frequent than every hour; dysuria, urgency, bladder spasm requiring local anesthetic (e.g. phenazopyridine)
3	Diarrhea requiring parenteral support; severe mucous discharge or blood discharge necessitating sanitary pads; abdominal distension (flat plate radiograph demonstrates distended bowel loops)	Frequency with urgency and nocturia hourly or more frequently; dysuria, pelvic pain, or bladder spasm requiring regular narcotic; gross hematuria with or without clot passing
4	Acute or subacute obstruction, fistula or perforation; gastrointestinal bleeding requiring transfusion, abdominal pain or tenesmus requiring tube decompression or bowel diversion	Hematuria requiring transfusion; acute bladder obstruction not secondary to clot passage, ulceration, or necrosis
5	Any toxicity that caused death	Any toxicity that caused death

Table S2 The results of univariable analyses of dosimetric parameters in patients without and with grade ≥2 AUT

**Dosimetric parameters , median (range)**	**Grade ＜2 (n=134)**	**Grade ≥2 (n=30)**	**OR**	**CI**	**p**
Bladder Volume (cm^3)	285.75 (35.20-1198.5)	284.10 (60.90-668.10)	0.999	0.996-1.001	0.296
Bladder Dmin	18.71 (2.41-173.08)	18.11 (9.79-32.71)	0.983	0.927-1.043	0.577
Bladder Dmax	54.16 (19.98-65.0)	54.08 (47.89-62.36)	0.976	0.901-1.057	0.548
Bladder Dmean	39.59 (26.23-60.6)	39.29 (33.84-47.06)	1.035	0.935-1.145	0.509
Bladder $D2cm^3$	53.29 (46.66-63.7)	53.33 (47.12-60.58)	0.941	0.840-1.054	0.290
Bladder D5% (Gy)	52.76 (46.23-61.6)	52.76 (46.70-54.26)	0.990	0.916-1.070	0.794
Bladder D10% (Gy)	52.25 (45.94-58.0)	52.40 (46.41-53.99)	0.951	0.825-1.097	0.493
Bladder D15% (Gy)	51.74 (45.65-55.1)	51.96 (45.77-53.76)	0.974	0.837-1.133	0.729
Bladder D20% (Gy)	50.90 (44.36-54.2)	51.04 (42.27-53.55)	0.997	0.926-1.073	0.941
Bladder D25% (Gy)	49.28 (41.90-53.6)	49.99 (42.20-53.33)	1.040	0.914-1.184	0.552
Bladder D30% (Gy)	47.46 (39.67-53.9)	47.86 (40.32-53.08)	1.051	0.922-1.197	0.458
Bladder D35% (Gy)	45.56 (37.57-51.7)	46.28 (37.93-52.74)	0.997	0.979-1.016	0.789
Bladder D40% (Gy)	43.73 (34.34-51.2)	44.88 (35.30-52.07)	1.117	1.001-1.247	**0.049**
Bladder D45% (Gy)	41.44 (29.04-50.6)	43.28 (33.32-51.40)	1.131	1.016-1.259	**0.024**
Bladder D50% (Gy)	39.35 (24.45-49.4)	41.43 (31.72-50.87)	1.141	1.026-1.269	**0.015**
Bladder D55% (Gy)	37.64 (20.79-47.6)	39.04 (30.19-49.85)	1.140	1.025-1.269	**0.016**
Bladder D60% (Gy)	35.93 (18.14-44.5)	37.46 (28.76-48.39)	1.179	1.056-1.316	**0.003**
Bladder D65% (Gy)	34.39 (16.19-43.2)	35.60 (27.33-46.40)	1.153	1.036-1.282	**0.009**
Bladder D70% (Gy)	32.61 (14.71-41.8)	33.53 (25.87-44.60)	1.119	1.012-1.237	**0.028**
Bladder D75% (Gy)	31.15 (13.10-40.8)	31.97 (24.29-43.42)	1.048	0.974-1.127	0.211
Bladder D80% (Gy)	29.64 (11.70-39.9)	30.35 (22.61-41.93)	1.058	0.970-1.154	0.205
Bladder D85% (Gy)	28.23 (10.58-39.0)	28.31 (20.80-40.36)	1.007	0.935-1.084	0.860
Bladder D90% (Gy)	26.61 (9.45-37.8)	25.67 (18.76-38.63)	1.020	0.944-1.101	0.620
Bladder D95% (Gy)	24.39 (8.13-36.4)	23.96 (15.52-36.94)	1.014	0.945-1.087	0.706
Bladder V5Gy (%)	100.00 (100.00-100.00)	100.00 (100.00-100.00)	NA	NA	NA
Bladder V10Gy (%)	100.00 (87.60-100.00)	100.00 (100.00-100.00)	0.995	0.962-1.029	0.762
Bladder V15Gy (%)	100.00 (68.90-100.00)	100.00 (95.60-100.00)	1.091	0.838-1.419	0.518
Bladder V20Gy (%)	99.70 (56.30-100.00)	99.85 (86.80-100.00)	0.988	0.959-1.017	0.402
Bladder V25Gy (%)	93.80 (49.30-100.00)	92.05 (72.80-100.00)	1.010	0.968-1.053	0.656
Bladder V30Gy (%)	78.75 (44.00-100.00)	82.20 (55.60-100.00)	1.015	0.984-1.046	0.354
Bladder V35Gy (%)	63.15 (39.40-98.0)	66.30 (40.70-99.10)	1.032	1.002-1.064	**0.037**
Bladder V40Gy (%)	47.35 (29.30-79.9)	54.05 (30.90-86.10)	1.072	1.030-1.117	**0.001**
Bladder V45Gy (%)	36.15 (18.60-58.1)	41.70 (17.50-68.10)	1.070	1.025-1.118	**0.002**
Bladder V50Gy (%)	23.20 (0.00-57.8)	25.90 (0.00-54.40)	1.001	0.973-1.030	0.926
Bladder V55Gy (%)	0.00 (0.00-15.30)	0.00 (0.00-13.50)	1.010	0.853-1.197	0.907

Abbreviations: OR: odds ratio; CI: confidence interval.

Table S3 The results of univariable analyses of dosimetric parameters in patients without and with grade ≥2 ALGIT

**Dosimetric parameters, median (range)**	**Grade <2 (n=130)**	**Grade ≥2 (n=34)**	**OR**	**CI**	**p**
Small Intestine Volume (cm^3)	522.2 (183.00-1169.40)	457.60 (314.80-1166.10)	0.999	0.996-1.001	0.187
Small Intestine Dmin (Gy)	1.98 (0.09-9.65)	1.93 (0.70-4.85)	1.084	0.787-1.455	0.597
Small Intestine Dmax (Gy)	52.36 (46.78-53.76)	52.69 (47.01-55.11)	1.074	0.907-1.295	0.425
Small Intestine Dmean (Gy)	23.73 (10.34-32.32)	24.88 (18.82-31.61)	1.097	0.997-1.217	0.067
Small Intestine D_{2cm^3} (Gy)	51.05 (45.51-52.53)	51.18 (45.73-52.62)	1.088	0.908-1.326	0.380
Small Intestine D5% (Gy)	47.75 (18.35-51.25)	48.48 (38.21-51.34)	1.105	0.978-1.280	0.149
Small Intestine D10% (Gy)	44.21 (32.53-50.48)	44.37 (31.81-50.84)	1.081	0.991-1.188	0.089
Small Intestine D15% (Gy)	39.37 (4.33-49.67)	41.46 (27.90-49.81)	1.081	1.009-1.166	**0.036**
Small Intestine D20% (Gy)	35.9 (23.58-49.42)	38.58 (25.75-48.18)	1.066	0.997-1.144	0.066
Small Intestine D25% (Gy)	33.23 (19.25-48.72)	35.95 (24.36-44.87)	1.064	0.996-1.140	0.070
Small Intestine D30% (Gy)	31 (7.93-52.71)	33.57 (21.29-41.60)	1.058	0.994-1.129	0.081
Small Intestine D35% (Gy)	28.84 (4.41-46.58)	31.20 (18.91-39.95)	1.065	0.999-1.142	0.063
Small Intestine D40% (Gy)	27.09 (1.96-44.84)	29.26 (17.28-38.17)	1.076	1.008-1.156	**0.036**
Small Intestine D45% (Gy)	25.23 (0.24-42.24)	27.10 (16.05-35.59)	1.074	1.007-1.154	**0.040**
Small Intestine D50% (Gy)	23.57 (0.78-37.4)	25.15 (14.92-32.85)	1.077	1.006-1.162	**0.044**
Small Intestine D55% (Gy)	21.76 (0.62-33.98)	23.31 (13.81-30.87)	1.071	1.000-1.158	0.066
Small Intestine D60% (Gy)	19.76 (0.51-31.7)	21.34 (11.33-28.14)	1.060	0.992-1.144	0.107
Small Intestine D65% (Gy)	17.45 (0.44-28.05)	18.49 (9.23-26.30)	1.050	0.983-1.130	0.168
Small Intestine D70% (Gy)	15.25 (0.38-41.88)	15.73 (2.16-24.67)	1.013	0.954-1.077	0.677
Small Intestine D75% (Gy)	12.85 (0.34-23.58)	13.01 (4.50-23.56)	1.023	0.957-1.097	0.504
Small Intestine D80% (Gy)	10.23 (0.29-21.39)	10.55 (3.23-22.07)	1.016	0.945-1.092	0.670
Small Intestine D85% (Gy)	7.38 (0.25-20.06)	6.63 (2.57-19.03)	1.015	0.936-1.098	0.717
Small Intestine D90% (Gy)	5.31 (0.17-18)	4.58 (2.15-16.19)	1.030	0.934-1.130	0.541
Small Intestine D95% (Gy)	3.75 (0.10-15.38)	3.42 (1.78-10.76)	1.039	0.904-1.182	0.574
Small Intestine V5Gy (%)	90.6 (92.00-100)	88.75 (73.60-100.00)	1.024	0.993-1.065	0.185
Small Intestine V10Gy (%)	80.4 (74.0-99.9)	80.55 (63.00-96.10)	1.020	0.992-1.055	0.201
Small Intestine V15Gy (%)	70.5 (26.60-95.6)	71.75 (49.60-92.30)	1.017	0.987-1.051	0.294
Small Intestine V20Gy (%)	59.35 (24.70-85.2)	62.70 (32.40-83.90)	1.019	0.988-1.053	0.246
Small Intestine V25Gy (%)	45.7 (17.60-73.1)	50.50 (22.50-68.40)	1.033	1.000-1.069	0.056
Small Intestine V30Gy (%)	31.85 (12.70-62.9)	38.25 (11.90-56.70)	1.040	1.006-1.077	**0.021**
Small Intestine V35Gy (%)	21.5 (7.20-52.8)	27.05 (7.20-45.90)	1.035	0.997-1.074	0.068
Small Intestine V40Gy (%)	14.9 (3.10-47.8)	17.70 (4.00-34.80)	1.030	0.986-1.076	0.174
Small Intestine V45Gy (%)	8.7 (1.00-39.6)	9.10 (1.70-24.80)	1.027	0.972-1.083	0.326
Small Intestine V50Gy (%)	2.3 (0.00-16.1)	2.80 (0.00-14.20)	1.069	0.963-1.182	0.196
Small Intestine V55Gy (%)	0 (0.00-0)	0.00 (0.00-0.00)	NA	NA	NA
Colon Volume (cm^3)	315.75 (90.70-1554.7)	254.55 (117.70-1261.10)	1.000	0.998-1.002	0.852
Colon Dmin (Gy)	2.19 (0.09-10.48)	2.12 (0.32-20.32)	1.110	0.912-1.389	0.272
Colon Dmax (Gy)	52.32 (26.87-53.93)	52.61 (46.30-57.42)	1.099	0.952-1.315	0.259
Colon Dmean (Gy)	25.34 (8.28-200.04)	26.14 (16.97-36.62)	1.002	0.965-1.027	0.852
Colon D_{2cm^3} (Gy)	50.83 (20.15-52.84)	51.32 (44.98-52.64)	1.095	0.951-1.323	0.298

Colon D5% (Gy)	49.46 (34.09-52.18)	50.18 (41.21-52.19)	1.118	0.969-1.321	0.158
Colon D10% (Gy)	47.07 (29.37-51.74)	49.10 (38.46-51.70)	1.129	1.006-1.295	0.059
Colon D15% (Gy)	44.8 (21.72-51.14)	47.48 (35.41-51.21)	1.136	1.032-1.272	**0.016**
Colon D20% (Gy)	42.03 (17.64-50.42)	44.71 (29.64-50.59)	1.086	1.007-1.183	**0.043**
Colon D25% (Gy)	39.11 (13.45-50.2)	40.72 (20.92-49.74)	1.059	0.996-1.134	0.079
Colon D30% (Gy)	35.91 (6.89-49.92)	38.13 (15.61-47.82)	1.054	0.998-1.118	0.069
Colon D35% (Gy)	32.32 (4.12-49.54)	35.93 (13.84-45.45)	1.063	1.009-1.126	**0.027**
Colon D40% (Gy)	28.44 (2.92-285.58)	32.62 (12.73-43.26)	1.003	0.983-1.020	0.659
Colon D45% (Gy)	25.47 (2.10-47.84)	28.24 (11.92-40.29)	1.076	1.020-1.139	**0.009**
Colon D50% (Gy)	23.13 (1.50-44.81)	24.89 (11.25-35.63)	1.051	0.997-1.112	0.072
Colon D55% (Gy)	20.74 (1.19-36.95)	22.31 (10.66-34.66)	1.066	1.005-1.135	**0.039**
Colon D60% (Gy)	18.32 (0.90-33.26)	20.18 (10.10-33.78)	1.069	1.005-1.142	**0.041**
Colon D65% (Gy)	16.34 (0.69-30.78)	18.02 (6.89-32.88)	1.063	0.995-1.139	0.077
Colon D70% (Gy)	14.57 (0.50-29.74)	15.73 (5.19-32.02)	1.062	0.992-1.141	0.088
Colon D75% (Gy)	12.52 (0.44-28.69)	14.15 (4.19-31.14)	1.055	0.985-1.132	0.131
Colon D80% (Gy)	10.04 (0.39-27.48)	11.05 (1.11-30.20)	1.032	0.961-1.107	0.375
Colon D85% (Gy)	6.73 (0.36-25.88)	7.29 (2.93-19.12)	1.026	0.946-1.110	0.522
Colon D90% (Gy)	5.17 (0.28-43.21)	4.95 (2.30-27.73)	1.004	0.924-1.076	0.905
Colon D95% (Gy)	3.8 (0.20-14.25)	3.59 (1.54-25.99)	1.055	0.924-1.195	0.390
Colon V5Gy (%)	90.65 (32.80-100)	89.80 (70.90-100.00)	1.022	0.986-1.070	0.281
Colon V10Gy (%)	79.85 (82.00-100)	81.30 (60.90-100.00)	1.025	0.994-1.062	0.140
Colon V15Gy (%)	68.5 (23.40-94.2)	72.40 (31.40-100.00)	1.025	0.995-1.058	0.117
Colon V20Gy (%)	56.4 (16.90-91.6)	60.25 (25.40-100.00)	1.029	1.000-1.061	0.058
Colon V25Gy (%)	46.25 (13.00-87.3)	50.45 (22.70-96.90)	1.029	1.000-1.062	0.056
Colon V30Gy (%)	38.6 (9.20-68.8)	42.95 (19.80-81.00)	1.038	1.006-1.074	**0.024**
Colon V35Gy (%)	31.15 (4.40-59.9)	36.70 (15.70-52.90)	1.038	1.003-1.076	**0.038**
Colon V40Gy (%)	24.05 (2.60-53.5)	25.80 (7.10-45.40)	1.042	1.002-1.085	**0.041**
Colon V45Gy (%)	14.3 (0.90-52.5)	19.55 (1.00-36.50)	1.040	1.000-1.083	**0.046**
Colon V50Gy (%)	3.75 (0.00-28.8)	6.25 (0.00-23.70)	1.061	0.992-1.135	0.079
Colon V55Gy (%)	0 (0.00-0)	0.00 (0.00-0.10)	NA	NA	NA
Rectum Volume (cm^3)	56.7 (18.20-181.3)	57.75 (24.40-107.10)	0.996	0.982-1.009	0.597
Rectum Dmin (Gy)	6.09 (1.83-36.06)	5.94 (2.32-33.29)	1.013	0.964-1.062	0.584
Rectum Dmax (Gy)	53.75 (47.22-64.77)	54.02 (47.34-61.55)	1.030	0.917-1.159	0.615
Rectum Dmean (Gy)	38.92 (27.78-50.49)	39.46 (32.21-47.41)	1.093	0.993-1.210	0.074
Rectum D_{2cm^3} (Gy)	52.18 (45.36-58.6)	52.26 (46.15-64.51)	1.073	0.938-1.236	0.309
Rectum D5% (Gy)	51.97 (45.39-56.86)	52.18 (45.84-53.73)	1.038	0.897-1.214	0.627
Rectum D10% (Gy)	51.25 (44.26-54.61)	51.53 (45.08-53.30)	1.042	0.898-1.224	0.596
Rectum D15% (Gy)	50.24 (43.18-53.45)	50.92 (44.23-52.88)	1.055	0.911-1.234	0.488
Rectum D20% (Gy)	49.13 (38.85-52.68)	50.24 (43.22-52.43)	1.069	0.932-1.239	0.358
Rectum D25% (Gy)	47.59 (34.08-52.21)	48.90 (42.08-51.90)	1.084	0.956-1.244	0.226
Rectum D30% (Gy)	46.17 (31.08-51.98)	47.62 (40.86-51.69)	1.100	0.979-1.247	0.122
Rectum D35% (Gy)	44.53 (29.19-51.74)	45.93 (39.63-51.41)	1.116	1.003-1.251	**0.049**
Rectum D40% (Gy)	42.94 (10.25-51.49)	44.06 (37.92-50.96)	1.110	1.012-1.230	**0.036**
Rectum D45% (Gy)	41.42 (26.44-51.25)	42.40 (35.25-50.34)	1.108	1.014-1.217	**0.027**

Rectum D50% (Gy)	39.78 (25.15-51)	41.05 (32.87-49.96)	1.104	1.017-1.205	**0.022**
Rectum D55% (Gy)	38.34 (23.39-50.76)	39.81 (30.88-49.83)	1.100	1.019-1.195	**0.018**
Rectum D60% (Gy)	36.96 (21.31-377.33)	38.71 (29.31-49.58)	1.000	0.972-1.012	0.968
Rectum D65% (Gy)	35.68 (19.55-50.26)	37.31 (27.26-48.85)	1.088	1.012-1.175	**0.026**
Rectum D70% (Gy)	34.27 (17.84-49.98)	36.36 (24.32-47.76)	1.078	1.007-1.161	**0.037**
Rectum D75% (Gy)	32.96 (13.28-49.66)	34.21 (21.60-46.23)	1.056	0.992-1.129	0.097
Rectum D80% (Gy)	31.38 (6.22-49.22)	31.94 (13.28-44.09)	1.033	0.977-1.098	0.272
Rectum D85% (Gy)	29.32 (3.96-48.6)	30.15 (7.25-41.66)	1.030	0.979-1.090	0.269
Rectum D90% (Gy)	26.69 (2.96-47.56)	28.05 (4.88-38.80)	1.025	0.981-1.075	0.281
Rectum D95% (Gy)	19.87 (2.40-45.71)	19.70 (1.00-34.65)	0.998	0.960-1.037	0.901
Rectum V5Gy (%)	100 (82.00-100)	100.00 (89.70-100.00)	0.976	0.865-1.127	0.707
Rectum V10Gy (%)	98.65 (77.00-100)	98.30 (82.00-100.00)	0.998	0.927-1.087	0.953
Rectum V15Gy (%)	96.9 (73.70-100)	97.35 (79.20-100.00)	1.012	0.949-1.091	0.724
Rectum V20Gy (%)	95.05 (8.30-100)	95.60 (76.90-100.00)	1.024	0.982-1.083	0.357
Rectum V25Gy (%)	91.45 (50.60-100)	91.45 (68.80-100.00)	1.024	0.986-1.072	0.254
Rectum V30Gy (%)	83.55 (32.60-100)	85.20 (57.70-100.00)	1.028	0.996-1.065	0.112
Rectum V35Gy (%)	67.3 (23.90-100)	72.45 (7.30-98.90)	1.022	0.995-1.050	0.116
Rectum V40Gy (%)	49.1 (5.70-99.8)	54.15 (33.50-88.20)	1.025	1.000-1.050	**0.048**
Rectum V45Gy (%)	33.9 (6.70-96.3)	37.15 (2.20-78.10)	1.013	0.990-1.037	0.265
Rectum V50Gy (%)	16.45 (0.00-69.6)	21.50 (0.00-49.10)	1.019	0.992-1.046	0.168
Rectum V55Gy (%)	0 (0.00-8.8)	0.00 (0.00-3.50)	1.030	0.479-1.612	0.902

Abbreviations: OR: odds ratio; CI: confidence interval.

Table S4 Dosimetric parameters of the PTV

Dosimetric parameters	45Gy group (n=39)	50.4Gy group (n=125)
D2% (cGy), medium (range)	4780.0 (4702.9-4996.7)	5381.4 (5167.8-6524.0)
D50% (cGy), medium (range)	4674.4 (4604.2-4766.7)	5250.3 (5039.1-5463.0)
D95% (cGy), medium (range)	4500.0 (4394.8-4593.3)	5028.9 (4607.0-5113.2)
D98% (cGy), medium (range)	4439.0 (4296.2-4552.2)	4963.7 (4473.1-5063.1)
